# Post-Dilution on Line Haemodiafiltration with Citrate Dialysate: First Clinical Experience in Chronic Dialysis Patients

**DOI:** 10.1155/2013/703612

**Published:** 2013-12-03

**Authors:** Vincenzo Panichi, Enrico Fiaccadori, Alberto Rosati, Roberto Fanelli, Giada Bernabini, Alessia Scatena, Francesco Pizzarelli

**Affiliations:** ^1^Nephrology and Dialysis Unit, Versilia Hospital, 55034 Lido Camaiore (Lucca), Italy; ^2^Nephrology and Dialysis Unit, Parma Hospital, 43100 Parma, Italy; ^3^Nephrology and Dialysis Unit, Lucca Hospital, 55100 Lucca, Italy; ^4^Nephrology and Dialysis Unit, SM Annunziata Hospital, 50012 Florence, Italy

## Abstract

*Background*. Citrate has anticoagulative properties and favorable effects on inflammation, but it has the potential hazards of inducing hypocalcemia. Bicarbonate dialysate (BHD) replacing citrate for acetate is now used in chronic haemodialysis but has never been tested in postdilution online haemodiafiltration (OL-HDF). *Methods*. Thirteen chronic stable dialysis patients were enrolled in a pilot, short-term study. Patients underwent one week (3 dialysis sessions) of BHD with 0.8 mmol/L citrate dialysate, followed by one week of postdilution high volume OL-HDF with standard bicarbonate dialysate, and one week of high volume OL-HDF with 0.8 mmol/L citrate dialysate. *Results*. In citrate OL-HDF pretreatment plasma levels of C-reactive protein and **β**2-microglobulin were significantly reduced; intra-treatment plasma acetate levels increased in the former technique and decreased in the latter. During both citrate techniques (OL-HDF and HD) ionized calcium levels remained stable within the normal range. *Conclusions.* Should our promising results be confirmed in a long-term study on a wider population, then OL-HDF with citrate dialysate may represent a further step in improving dialysis biocompatibility.

## 1. Introduction

There is evidence that in chronic dialysis patients haemodiafiltration (HDF) induces better control of phosphatemia [1, 2] and lower *β*2-microglobulin (*β*2-m) blood levels [2, 3] with observed better clinical outcomes [4, 5]. The effect on *β*2-m has been related not only to HDF efficiency in toxin removal [6] but also to the potentials of convective treatments in slowing down inflammation. As a matter of fact, HDF has been associated with reduction of proinflammatory cytokines [7, 8] and circulating proinflammatory cells [9].

However, in online HDF (OL-HDF) the small amount of acetate of standard bicarbonate dialysate may itself induce inflammation due to the large amount of fluids infused into the patient blood stream [10]. Hence, search for optimal biocompatible dialysis treatments is still an unsolved goal.

Ahmad et al. were the first to use in bicarbonate dialysate an acid concentrate made by replacing citric acid for acetic acid [11]. The final dialysate had a citrate level of 2.4 mEq/L (0.8 mmol/L). This citrate-enriched “acid concentrate” is commercially available and is now widely used in haemodialysis in different countries, particularly USA [12–16].

The citrate in the dialysate crosses the dialyser membrane to chelate calcium in the blood flowing within the dialyser and the venous tubing, thus impairing the clotting process to bring about regional anticoagulation. By doing so, however, citrate has the potential drawback of inducing hypocalcemia, and this side effect may be amplified in OL-HDF, due to large amounts of dialysate infused. The rationale for citrate relies not only on anticoagulative properties [17] but also on its possible favorable effect on dialysis-induced inflammation [18–22]. Thus, OL-HDF with citrate dialysate may represent a further step in improving dialysis biocompatibility, providing that it does not induce clinically relevant hypocalcemia.

With this background, in this small, pilot study we wanted to evaluate safety, feasibility, and anti-inflammatory capability of high volume postdilution OL-HDF performed with citrate-enriched dialysate infusate.

## 2. Materials and Methods

### 2.1. Study Design

This is a pilot, short-term study. Patients underwent one week (3 dialysis sessions) of bicarbonate dialysis with 0.8 mmol/L citrate-enriched dialysate (citrate HD, phase A), sequentially followed by one week of OL-HDF with standard bicarbonate dialysate (standard OL-HDF, phase B), and then one week of OL-HDF with citrate-enriched dialysate (citrate OL-HDF, phase C).

### 2.2. Patients

Thirteen chronic dialysis patients of Florence and Versilia Nephrology Units were enrolled.

Inclusion criteria were AS follows: age >18 years and <80 years and a vascular access suitable for easily obtaining a blood flow >300 mL/min; patients affected by chronic liver disease, active neoplastic or inflammatory disease were excluded as well as patients receiving immunosuppressive or anti-inflammatory drugs.

The study was approved by the Local Ethical Committee of the two hospitals, and all patients signed a written consent form.

### 2.3. Dialysis Parameters

Citrate dialysate (Citrasate, Advanced Renal Technologies Inc., Washington, USA) contained 0.8 mmol/L of citric acid. Acetate concentration was 0.3 mmol/L in citrate dialysate and 2.5 mmol/L in standard bicarbonate dialysate. Ca dialysate concentration was always 1.5 mmol/L. Sodium and potassium were, respectively, 137 mmo/L and 2.0 mmo/L. Ultrapure dialysate was used in all the dialysis sessions.

Anticoagulation was performed as LMWH in all patients; dalteparin was administered starting dialysis at the dose of 60.4 ± 11.2 IU/kg, and the dose was not changed during the study period.

Patient-related (body weight, blood Pressure, BP, and heart rate, HR) and monitor-related parameters were collected during each session of all treatments. Dialysis prescription is reported in Table 1. Kt/V, as a proxy of treatment efficacy, was continuously monitored by a biosensor based on UV mass spectrometry (ADIMEA, B Braun Avitum, Melsungen Germany). This biosensor is integrated in the dialysis monitor utilized in this study, and it has been validated for use both in HD and in HDF [23, 24].

### 2.4. Laboratory

Serum total calcium, ionized calcium (Ca^++^), and bicarbonate were determined at baseline (T0), one hour (T1), 2 hours (T2), and at the end (T4) of each treatment, while plasma samples for citrate and acetate measurements were drawn at T0-T2-T4. Activated partial thromboplastin time (aPTT), *β*2-m and CRP were checked at T0-T4. End-treatment *β*2-m, values were normalized for haematocrit according to the Bergstrom formula [25]. Commercially available UV test kits for enzymatic spectrophotometric analysis were used for citrate (Enzyplus EZA785+, Biocontrol, Italy) and acetate (Enzyplus EZA811+, Biocontrol, Italy) measurements on serum and ultrafiltrate/dialysate samples. Citrate and acetate determinations were centrally performed in Nephrology laboratory of Parma University. Other routine parameters were analyzed by standard methods.

### 2.5. Statistics

Continuous data are presented as mean ± standard deviation (SD). Differences between mean values were evaluated by paired-samples *t*-Test or by Wilcoxon signed ranks test for not normally distributed data. Analysis of variance (ANOVA) for multiple comparisons was used to analyse differences between groups. Spearman correlation coefficient was calculated for correlation assessments between variables. A *P* value less than 0.05 was considered statistically significant.

## 3. Results

Parameters of dialysis prescription (Table 1) were satisfied during the three experimental procedures. Relevant hydraulic pressures achieved in the three phases of the study are reported in Table 2.

### 3.1. Safety

No adverse effect was observed. Hypotensive episodes were globally very low and similar over the three study phases. Average BP values were almost super-imposable among various treatments, being systolic/diastolic figures 128 ± 22/73 ± 13 mmHg, 129 ± 25/74 ± 14 mmHg, and 130 ± 21/72 ± 16 mmHg in HD, standard HDF, and citrate HDF, respectively (*P* = NS), the same for HR, which was 77 ± 12 beats/min, 77 ± 14 beats/min, and 77 ± 13 beats/min in the 3 study phases, respectively [*P* = NS].

At the beginning of treatments plasma Ca^++^ was identical in the three phases (Table 3). At variance with standard OL-HDF, plasma Ca^++^ did not increase during both citrate treatments with values remaining always within the normal range; accordingly, Ca^++^ values were significantly higher at the end of standard OL-HDF with respect to citrate OL-HDF. Total calcium values (Table 3) progressively increased during all treatments but with different slope, such as absolute values of total calcium that were significantly higher at the end of standard OL-HDF in comparison to citrate OL-HDF. As for coagulation (Table 3), aPTT values were longer at the end of citrate OL-HDF than in standard OL-HDF, the difference being highly statistically significant although of minor clinical relevance.

### 3.2. Dialysis Efficiency

Kt/V was 1.35 ± 0.27, 1.68 ± 0.31, and 1.69 ± 0.28 in citrate HD, standard OL-HDF, and citrate OL-HDF, respectively, with highly significant (*P* < 0.001) differences among HD and OL-HDF treatments. The average infusion volumes obtained were very high, for example, 21.5 ± 2.2 liters in standard OL-HDF and 21.9 ± 1.9 liters in citrate OL-HDF (*P* = NS). Dialyzer hydraulic pressure profiles were similar in both OL-HDF treatments (Table 2). As expected, HDF treatments consistently reduced plasma *β*2m values, while HD did not (Table 3). *β*2m clearance was quite similar in both HDFs, being 83.8 ± 6.3 mL/min in standard OL-HDF and 83.0 ± 6.8 mL/min in citrate OL-HDF (*P* = NS). Plasma levels of phosphate, bicarbonate, Na, and K did not differ either at the beginning or at the end of treatments.

### 3.3. Biocompatibility

At the beginning of treatments, *β*2m plasma values were significantly higher in HD than in both OL-HDF treatments. What matters more here is the finding that *β*2m plasma values were highly significantly lower at the beginning of citrate OL-HDF with respect to standard OL-HDF. This datum is confirmed by the significantly lower levels of CRP at the beginning of citrate OL-HDF versus standard OL-HDF (Table 3). These analyses were made keeping into account that baseline values of the first treatment of the week actually belonged to the treatment performed in the previous week.

### 3.4. Citrate and Acetate Handling

**Figure 1:**
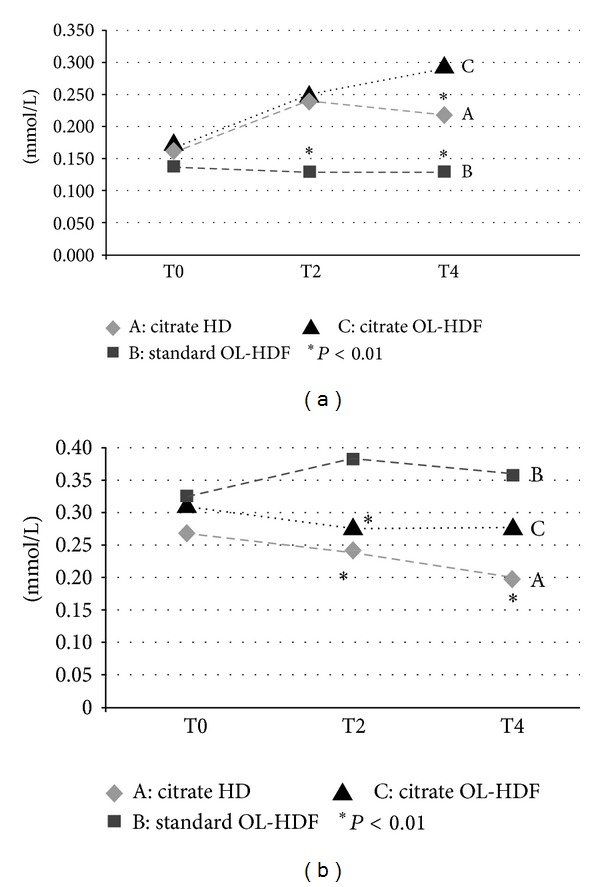
(a) Plasma levels of citrate during treatments. (b) Plasma levels of acetate during treatments.

As expected, plasma levels of citrate varied according to the presence or not of citrate in the dialysate. Citrate plasma levels reached zenith at T2 in HD to become then stable, while they progressively increased during citrate OL-HDF with values at T4 significantly higher than in citrate HD (Table 3 and Figure 1(a)). Zenith citrate plasma levels were much lower than the threshold values considered as potentially toxic [26]. Citrate was rapidly metabolized in the intertreatment period, and all subjects had baseline plasma values superimposable, irrespective of treatments.

Also plasma acetate levels reflect the amount of this buffer contained in the dialysate. Only in standard OL-HDF plasma acetate levels significantly progressed throughout treatment, while figures remained stable, or even reduced, in the other two procedures (Table 3 and Figure 1(b)). Since in our study patients were observed in clinical routine, thus with no control on diet nor advice to fast, baseline plasma acetate levels, proxy of acetate body production, were higher than figures observed in other studies where fasting was mandatory [10].

## 4. Discussion 

In this small, pilot study we have observed that, in comparison with standard OL-HDF, OL-HDF performed with a new dialysate substituting citrate for acetate may bring about a lower inflammation, as heralded by the lower pretreatment *β*2m and CRP serum levels for the same treatment efficiency, and a light “anticoagulant” effect. As for safety, during both citrate techniques, for example, HD and OL-HDF, plasma calcium levels were stable within normal range. However, this is an acute study while safety ought to be challenged against time. It remains that, to the best of our knowledge, this is the first study performed in chronic dialysis subjects aimed at exploiting advantages and hazards of citrate dialysate in high-volume convective therapies.

Even if the two OL-HDF treatments challenged in this study showed similar urea and *β*2-m removal and no difference in hydraulic pressures inside the dialyzer, pretreatment plasma levels of *β*2-m and CRP were significantly lower with citrate OL-HDF. Since treatments were performed one week apart on the same patients utilizing the same dialyzer and the same amount of liters infused, we deem that the result may be explained by a direct anti-inflammatory action of citrate at low doses. In addition to this, citrate concentrate has lower levels of acetate than standard bicarbonate concentrates and it has demonstrated lower inflammatory effects linked to acetate-free dialysate [10].

In our study the anti-inflammatory effect of HDFs was achieved within few days, while in other studies pretreatment *β*2-m plasma levels were reduced, if any, only after several months of convective treatments [3, 4]. The difference might be due to the much higher volumes exchanged in our study. Aside from the volume effect, the anti-inflammatory effect of citrate is elicited *in vitro* within 48 hours of incubation [27] while favorable effects of acetate-free dialysate are achieved within few hours in both *in vitro* [28, 29] and *in vivo* studies [10].

The anticoagulant effect of citrate is due to the low-calcium environment in the blood; many important enzymatic steps of the coagulation cascade are in fact calcium dependent and citrate acts by chelating ionized calcium with the formation of Ca-citrate complexes [30]. To obtain regional anticoagulation, citrate is added in predilution either directly [31] or in the reinfusion fluid in continuous renal replacement therapies [32]. Otherwise, as in our study, citrate can be added to dialysate by utilizing a concentrate acidified with citric acid [11]. In this latter case, citrate crosses the dialyzer membrane to reach the blood, thus impairing coagulation in blood extracorporeal circuit. Different mechanisms contribute to keep low systemic citrate levels during regional anticoagulation. First of all, citrate has a short half-life, for example, 49 min, being rapidly metabolized to CO_2_ and water when it enters the tricarboxylic-acid cycle in the liver, and to a lesser extent in the renal cortex and skeletal muscle [33]. Since citrate is metabolized as citric acid, its metabolism consumes hydrogen ions, produces bicarbonate, and may lead to an increase in blood pH [34]. Total metabolic body clearance of citrate in healthy subjects receiving short-term loads (0.5 mmol/Kg/hour) is about 700 mL/min. Although systemic metabolic clearance is reduced by at least 50% in patients with liver failure, safety of regional citrate anticoagulation has been recently demonstrated also in liver transplant patients and in liver failure [32, 35]. Secondly, calcium-citrate complexes are efficiently removed during renal replacement therapy. In fact, both diffusive and convective clearance markedly reduce the citrate load to the patients (up to 75% reduction), thus increasing the feasibility and tolerance of citrate-based protocols [33, 34]. In our patients citrate plasma levels at the end of treatments were lower than 0.29 mmol/L, well below the values of 0.85 mmol/L, considered the upper limit of safety even for critically ill patients [26].

With systemic citrate levels being exquisitely low in our patients, it comes as no surprise that their plasma calcium values (total and ionized) did not decrease during citrate dialysis sessions and instead remained stable within the normal range. Moreover, many protocols for citrate hemodialysis utilize calcium-free dialysate to prevent precipitation of calcium-citrate complexes with its attendant reduction of the anticoagulant effect of citrate. This was not the case in our protocol, being dialysate Ca concentration 1.5 mmol/L, thus preventing the negative effects on serum calcium levels of Ca-free dialysate.

We acknowledge several limitations of this study. Dialysis sequences were not randomized; thus we cannot exclude with certainty the bias of carry over effect.

Second, but not less relevant, our study was too short to affirm safety of citrate OL-HDF. We can only say that from the stand point of plasma calcium fluctuations, citrate OL-HDF was as safe as citrate HD being the latter an available option extensively implemented in several countries [12–16].

In conclusion, in this small, acute study we challenged for the first time a new acid concentrate containing citrate in OL-HDF with an average postdilution exchange of 21 liters per treatment.

This new dialysis technique did not generate hypocalcemia and was associated with lesser dialysis-induced inflammation. Randomized, prospective, long-term studies on a wider population are decisively necessary to confirm the encouraging results of this pilot study.

## Figures and Tables

**Table 1:** Dialysis prescription.

	Citrate HD-phase A	Standard OL-HDF-phase B	Citrate OL-HDF-phase C
Membrane	Low flux *α* polysulfone 2.0 m^2^	High flux *α* polysulfone 2.3 m^2^	High flux *α* polysulfone 2.3 m^2^
Qb	≥300 mL/min	≥300 mL/min	≥300 mL/min
Qd	500 mL/min	500 mL/min	500 mL/min
Qinf*		6 L/h in function of MIDP	6 L/h in function of MIDP
Treatment time	240 ± 15 min	240 ± 15 min	240 ± 15 min

*Modulated according to the maximum inlet dialyzer pressure [MIDP] set at 650 mmHg according to manufacturer.

Qb: blood flow.

Qd: dialysate flow.

Qinf: infusion flow.

**Table 2:** Achieved operating dialysis monitor pressures.

	Citrate HD-phase A		Standard OL-HDF-phase B		Citrate OL-HDF-phase C	
	T0	T4	T0	T4	T0	T4
TMP (mmHg)	78.5 ± 31.4	56.9 ± 37.7	97.4 ± 26.18	127.2 ± 35.5	103.9 ± 43.5	157.5 ± 75.2
MDIP (mmHg)	276.9 ± 24.5	310 ± 51.8	333 ± 44.3	426.2 ± 103	337.4 ± 56.6	429.4 ± 131.5

TMP: 3 point trans membrane pressure; MDIP: Maximum dialyzer inlet pressure.

No statistically significant differences between phase B and C.

T0 starting dialysis.

T4 ending dialysis.

**Table 3:** Laboratory data analysis.

	Citrate HD-phase A				Standard OL-HDF-phase B				Citrate OL-HDF-phase C			
	T0	T1	T2	T4	T0	T1	T2	T4	T0	T1	T2	T4
Ca^++^ (mmol/L)	1.07 ± 0.06	1.05 ± 0.04**	1.05 ± 0.04**	1.05 ± 0.03**	1.08 ± 0.05	1.16 ± 0.05	1.17 ± 0.05	1.21 ± 0.008	1.08 ± 0.06	1.07 ± 0.04**	1.07 ± 0.03**	1.06 ± 0.06**
Ca tot (mmol/L)	8.7 ± 0.5	9.0 ± 0.3	9.1 ± 0.3	9.5 ± 0.4	8.9 ± 0.4	9.5 ± 0.3	9.6 ± 0.4	10.2 ± 0.6	8.6 ± 0.6	9.1 ± 0.4	9.3 ± 0.5	9.4 ± 0.4**
Citratemia (mmol/L)	0.15 ± 0.03		0.22 ± 0.05	0.22 ± 0.05^§§^	0.13 ± 0.02		0.13 ± 0.02	0.13 ± 0.02	0.17 ± 0.07		0.24 ± 0.07**	0.28 ± 0.06**
Acetatemia (mmol/L)	0.25 ± 0.13**		0.26 ± 0.11**	0.20 ± 0.06**	0.32 ± 0.13		0.38 ± 0.08	0.35 ± 0.09	0.31 ± 0.18		0.27 ± 0.12**	0.28 ± 0.15**
*β*2 (mg/L)	36.3 ± 8.0^∗∗§§^			40.2 ± 14^∗∗§§^	28 ± 5.5			6.5 ± 2.7	25.7 ± 4.7**			5.7 ± 2.1
CRP (mg/L)	8.0 ± 9.5				7.7 ± 5.9				5.9 ± 5**			
Aptt (sec)	31.6 ± 8.2			41.9 ± 9.7	31.1 ± 3.6			39.0 ± 6.5	34.92 ± 11.6			42.2 ± 10.7**

**P < 0.01 versus phase B; ^§§^ *P* < 0.01 versus phase C.

T0 starting dialysis.

T1 after one hour of dialysis.

T2 after two hours of dialysis.

T4 ending dialysis.
